# Demographic Transition in India: An Evolutionary Interpretation of Population and Health Trends Using ‘Change-Point Analysis’

**DOI:** 10.1371/journal.pone.0076404

**Published:** 2013-10-18

**Authors:** Srinivas Goli, Perianayagam Arokiasamy

**Affiliations:** 1 Department of Development Studies, Giri Institute of Development Studies (GIDS), Lucknow, Uttar Pradesh, India; 2 Department of Development Studies, International Institute for Population Sciences (IIPS), Mumbai, Maharashtra, India; CUNY, United States of America

## Abstract

**Background and Rationale:**

Lack of a robust analytical tool for trend analysis of population and health indicators is the basic rationale of this study. In an effort to fill this gap, this study advances ‘Change-Point analyzer’ as a new analytical tool for assessment of the progress and its pattern in population and health indicators.

**Methodology/Principal Findings:**

The defining feature of ‘change-point analyzer’ is that, it detects subtle changes that are often missed in simple trend line plots and also quantified the volume of change that is not possible in simple trend line plots. A long-term assessment of ‘change-point analyses’ of trends in population and health indicators such as IMR, Population size, TFR, and LEB in India show multiple points of critical changes. Measured change points of demographic and health trends helps in understanding the demographic transitional shifts connecting it to contextual policy shifts. Critical change-points in population and health indicators in India are associated with the evolution of structural changes in population and health policy framework.

**Conclusions:**

This study, therefore, adds significantly to the evolutionary interpretation of critical change-points in long-term trajectories of population and health indicators vis-a-vis population and health policy shifts in India. The results have not only helped in reassessing the historical past and the current demographic transition trajectory but also advanced a new method of assessing the population and health trends which are necessary for robust monitoring of the progress in population and health policies.

## Introduction

All the societies, at one time or another, move from a near-equilibrium condition of high mortality and high fertility towards a presumed low-fertility and low-mortality equilibrium termed as ‘demographic transition’ the process pioneered by Notestein [1]. Since 1950, demographic transition has occupied center stage in demographic analyses and, therefore, the progress in demographic transition need to be understood and interpreted correctly [2]. The contemporary theory of population change revolves around the concept of the classical demographic transition model enunciated by Notestein [1]. However, a perfect portrayal of demographic transition is required to investigate the impact of demographic changes on social, economic and political structures of nations. A largely acceptable characterization of demographic transition consists of five components: mortality decline, natural increase in population size, fertility decline, urbanization and population aging. For instance, the conception of a shift from a regime of negligible population growth characterized by high birth and death rates to one of equally little growth based on low birth and death rates, during which there is a rapid increase in numbers due to the demographic gap, the lag of fertility decline behind the mortality decline. Although, this conception is only a rough generalization, it has such wide applicability that it has become a central axis of conceptualization about population trends [1], [3]–[7].

Numerous studies have assessed demographic transition in terms of fertility, mortality and health trends and transition on the global scale [6], [8]–[21]. A growing number of studies in India had also assessed the trends and transition in population health indicators [5], [22]–[36]. However, all these studies have used basic trend line plots, control charts or descriptive tables with annual or decadal changes to determine transition points in population and health indicators. To our knowledge and to date, there are no studies across the globe or in India that attempted an evolutionary interpretation of long-term trends in population and health indicators through more sophisticated tools and techniques, which have properties to estimate, and determine critical change-points and multiple changes, and affirm its statistical significance.

This study adopted an innovative procedure is termed as ‘change-point analyses’ to study, the long-term trends in population and health indicators in India and states. The main distinguishing factor between ‘change-point analyses’ and simple trend line plots or charts is that, simple trend line plots are in general better at identifying isolated uneven points and major changes whereas a ‘change-point analyses’ can also detect modest changes that are frequently unobserved by control charts. Moreover, the simple trend line plots or charts cannot quantify the change with its statistical significance including confidence levels. Estimation of confidence levels of a change is essential to validate and determine the robustness of a change that appears in graphical plots and charts. While examining historical data, particularly when data sets are large ‘change-point analyses’ is favoured, as it is scientifically robust than the control charts in terms of identifying the pattern of trend. The other key advantage of a ‘change-point analyses’ is that it controls the change-wise error rate; therefore, each change identified is possibly the truest. Simple trend line plots or ‘control charts’ or descriptive tables of annual or decadal change do not control the point wise error rate and, therefore, may provide an inaccurate assessment of demographic trends. When analyses are performed in large numbers of data points, several points can go beyond the set limits even when no change may have occurred. The main advantage of ‘change point analyses’ is: it is simple to apply and construe, especially for large data sets and when multiple changes may have occurred [37].

Through ‘change-point analyses’ of the population and health indicators, this paper addresses five questions: 1) did change occur at all? 2) did more than one change occur? 3) when did the changes occur? 4) with what confidence level did the change occur and 5) what demographic expositions emerge from the observed change-points? By assessing these five questions in Indian context, this study attempt, to identify the commencement of critical changes and multiple change points in various demographic indicators over a long period of demographic transition. Implicitly, this study is an effort to integrate demographic change with population and health policy shifts and other historical events; and thus, assesses the temporal dimension of the demographic progress. With the determination of critical change-points and their time points, this study innovate to more accurately interpret the accounting factors of progress in demographic and health transitions. To establish the causal relationship, this study compares historical trends and pattern of fertility, mortality and population size by relating the trend to time trajectories of population policies and development strategies as drivers of change.

## Data and Methods

This study attempted to investigate long-term (around 150 years) demographic trend data for various reasons. The long term trends observed better than short intervals of time because of the defectiveness of the available statistical material, where specific annual totals and rates in births and deaths do not have much significance. While examining and establishing trends in health and demographic transition, one hundred and fifty years constitute a convenient and significant stretch of time for identifying a pattern and progress. This has been proved adequate in the case of Western Europe, where the demographic cycle began around 1750 and lasted until the depression–a period of more than 200 years [4], [38]. However, the major impediment of historical analyses of demographic trends in India is the absence of good quality of data as the vital registration system is far from functioning well [26]. It is well known that, in India, the coverage of vital registration system is far from being complete to provide reliable estimates of population and health indicators for the period until 1970 [35]. This study, therefore, uses myriad sources of data: Census data, indirect estimates, survey based and other official estimates to assess the long-run trends of population size, fertility and mortality indicators. The detailed description of different data sources used in this study is presented below.

### Mortality and Fertility Indicators

The historical Total Fertility Rate (TFR) and Infant Mortality Rate (IMR) data in pre-1971 India is constructed based on indirect estimates of various sources: 1) Data on TFR and Life Expectancy at Birth (LEB) during 1951–1971 are based on Rele estimates [24]. The Rele estimates are considered to be robust compared to other estimates of fertility and mortality prior to the Sample Registration System (SRS) estimates. 2) The LEB data for the pre-1951 period was obtained from estimates provided in Mitra [39]. Mitra provided LEB estimates based on Registrar General of India (RGI) and Census during 1872–1941. The estimates of total LEB are averages of male-female LEB [39]. However, we don’t have reliable TFR estimates for the pre-1951 period. The recent data on LEB, fertility and mortality indicators are taken from the SRS, available on an annual basis [44]. More recent data on LEB were adopted from the estimates of United Nation Development Programme [40].

### Population Size

The data on population size prior to 1955 were based on estimates of Mukherjee [41]. We have chosen Mukherjee estimates [41] over Davis [3]; Ghosh [22] and Mahalanobis and Bhattacharya [42] etc. because his estimates of population totals from, 1856 to 1951 followed a uniform method, which are comparable over time. However, the recent statistics on population size obtain from Reserve Bank of India (RBI) [43]. Mukherjee [41] and RBI [43] both estimated population totals for five year intervals based on linear interpolation between Censuses [44]; therefore, both these estimates are comparable over a long-term period. However, the population totals for the year 2011 is obtained from provisional Census results of 2011 [45].

### Change-Point Analyses

In this paper, we have used ‘change-point analysis’ technique to assess the critical and multiple change-points over the long-term population and health trends of India. A general usage of word ‘change-point’ indicates ‘the time at which a change began to occur’ [37]. However, a critical change-point is the point where a major shift in the trend is recognized. The change-points highlighted in this paper are critical change-points. A critical change will not appear immediately, rather an accumulative growth or decline will results into a critical change for a given indicator. However, the time period for resulting a change into a critical change depends on sensitivity nature of the indicators to its external factors. For example, in comparison to population size, a critical change in child mortality rates and for some extent the fertility rates yield in lesser time. For each change, ‘change-point analyzer’ provides detailed information including volume of change, confidence levels and change-wise error rate during the trend period. “The procedure of performing a ‘Change-Point analyses’ is very flexible, as it can be carried out on all types of time series data as well as attribute data, data from non-normal distributions, ill-behaved and complaint data, and data with outliers”. However, the change-point analyses yield better results on long period data because on long period data, we can observe the pattern and time shifts more clearly [37].

**Figure 1:**
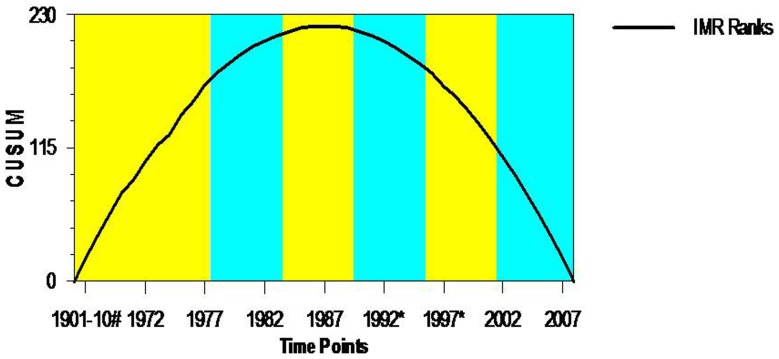
CUSUM Chart of IMR for Indian, 1901–2008.

There are several approaches to performing a trend analysis and change point analyses [37], [46]–[54]. However, we used the method proposed by Taylor for performing the ‘change-point analyses’ which generally use a combination of ‘Cumulative Sum’ (CUSUM) Charts and bootstrapping to detect changes [39]. “The outliers in any population data create additional distinction in the data making it more convoluted to detect a change. The ‘change-point analyses’ technique is more robust to such outliers. These estimates can be made even more robust by analyzing the ranks of values as an alternative of the values [37]. We have adopted the procedure of analyzing the ranks of the values instead of the values. Change-point analyses involve following procedures: the analysis begins with the construction of the CUSUM chart shown in Figure 1 and Appendix S1.

CUSUM plots are built by estimating cumulative sum based on the ranks of IMR data instead of actual IMR values. Let *IMR_1_, IMR_2_…., IMR_43_* represent the 43 data points. From this, the cumulative sums *S_1_, S_2_…., S_43_* are calculated. The procedure for estimating the CUSUM proposed by Taylor [54] is given below:

- The first step in estimating CUSUM is to estimate the average

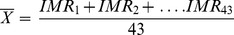

- In the second step one should begin the CUSUM at zero by setting S_0_ = 0. Then estimate the other CUSUM by adding the difference between the current value and the average to the previous sum, i.e. S_1_ = S_1-I_+X_1_−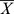 for *i* = 1, 2…, 43.

Here, the CUSUMs are not the CUSUMs of the values; instead, they are the CUSUMs of differences between the values and the average. “These differences sum up to zero so the CUSUM always ends at zero (S43 = 0)” [54]. The CUSUM chart in figure 1 appears to indicate that at least 1 and possibly 6 changes took place. However, the difficulty with CUSUM charts is that they require exceptional skill to understand correctly. Further, one cannot affirm convincingly that these changes took place? For this, the confidence levels of each change-point estimate can be used to understand the apparent change by undertaking a bootstrap analysis. Before undertaking the bootstrap analysis, an estimator of the amount of the change is required [54]. According to Taylor [54] one option, which works well in any case of the distribution and regardless of multiple changes, is S*_diff_* defined as:
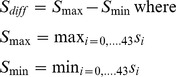



Following this, bootstrap analysis can be performed when the estimator of the amount of the change has been fixed [49], [54]–[55]. According to Taylor [54] a single bootstrap is performed through the following steps:

1. A bootstrap sample of 43 units can be generated by randomly reordering the original 43 values and denoted: 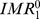, 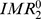 …… 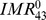. This is referred to as sampling without replacement [54].
2. Once the bootstrap sample is generated, based on this bootstrap sample, the bootstrap CUSUM is calculated and denoted by 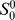, 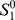, …,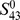.
3. In the next step, the difference of the bootstrap CUSUM is calculated by taking maximum, minimum, denoted by S*_max_*, S*_min_*, and S*_diff_*.
4. And the last step involves determining whether the bootstrap difference 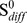 less than or more than the original difference.

The rationalisation behind generating the bootstrapping is that the bootstrap samples signify for the random reallocation of the data that mimic the behaviour of the CUSUM if no change has occurred. With the help of number of bootstrap samples, one can calculate how much S*_diff_* would vary if there is no change took place. This can be compared with the S*_diff_* value calculated from the data in its original order to determine if this value is consistent with what we would anticipate if no change occurred. The bootstrap CUSUM charts tend to stay closer to zero than the CUSUM of the data in its original order. This indicates that a change must have occurred [54].

Further a confidence level of the estimate is calculated. If one assumes that ‘N’ is the number of bootstrap samples performed and let X be the number of bootstraps for which 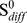 < S*_diff_*. Hence, the confidence level = 100 x/N%. Typically 90% or 95% confidence is required to determine that a significant change has occurred. Out of 1,000 bootstraps, 995 had 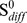 < S*_diff_*. This gives a confidence level = 100 x/N% = 99.5%; this indicates strong evidence that a change did, in fact, occur [54].

## Results

The results of ‘change-point analyses’ demonstrate trends and patterns in key population and health indicators of India: 1) IMR 2) Population size 3) TFR and 4) LEB. These four demographic indicators represent four critical components of demographic transition in their sequential order: mortality decline, increased rate of natural increase, fertility decline and increasing life expectancy or population aging [6]. The results are presented in accordance with the sequence of these four key components in the demographic transition process.

### IMR

IMR is the most sensitive and widely used summary measure to understand the overall progress in mortality decline and improvement in population health. Numerous demographic and public health research studies have overwhelmingly concluded that the decline in IMR is majorly the consequence of the complementary progress in modern health care and socioeconomic advancement. As indicated by Davis in the Change Response model, it is the first component of demographic transition [1], [4]. In the recent refreshing exposition of demographic transition, Dyson [6] believed “mortality decline is the crucial catalyst; once it occurs, the other four components necessarily follow, and most often sequentially because one component generates the next. Mortality decline leads to an increased rate of natural increase, which produces the conditions that cause fertility decline, which in turn leads to increase in life expectancy and population aging”.

Over the last more than half a century, India experienced a phenomenal decline in IMR. Nevertheless, India’s current level of IMR (54/1000 by 2008) is seen to be relatively higher compared to many developed and developing countries of the world. By analyses of historical trends of mortality, Chandrasekhar, Bhat *et al* and Dyson *et al* [5], [28], [38] have documented the phenomenal decline of infant mortality rate from 1921–which accelerated sharply after 1947. Until the late 1970s, the IMR showed a declining trend but with considerable discrepancies, followed by a continuous decline in post-1970–which led to the steady increase in natural growth rate and acceleration in the population size. However, virtually all previous studies that assessed trends in IMR through simple trend line plots. As trend lines are methodologically imperfect tools, they have not useful to detect and quantify the critical change-points in understanding long-term trends in mortality transition based on IMR.

**Figure 2:**
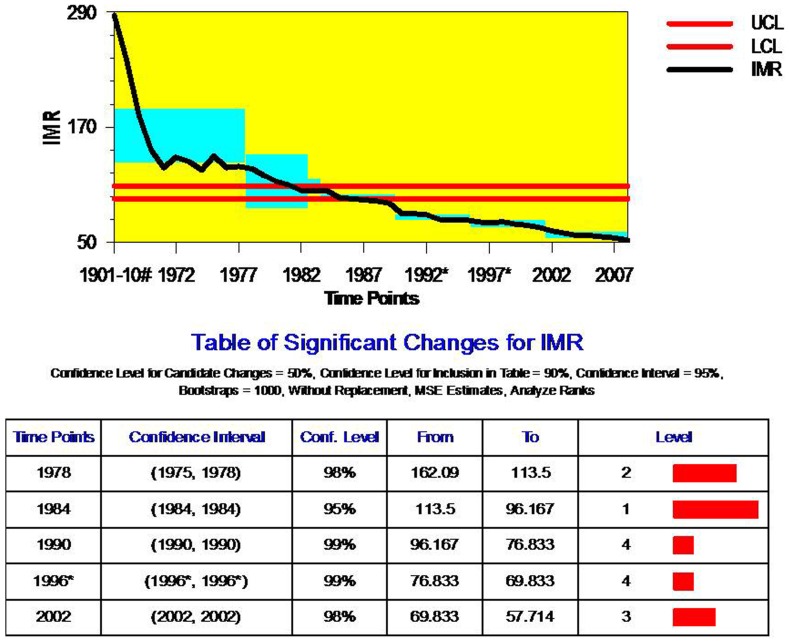
Change Point Analyses of Trend in Infant Mortality Rate for India, 1901–2008. Note: 1. UCL- Upper side of Confidence Level; LCL- Lower side of Confidence Level. 2. Level- Confidence Level. *Source:* 1. ^#^Rele, 1987. 2. Office of Registrar General of India, 1971–2008. 3. * Excludes Jammu & Kashmir due to non-receipt of returns.

Figure 2 presents a plot of IMR trend and change-point estimates during 1901–2008. During the past, more than 100 years, results indicate five critical change-points mostly in the last 30 years (1978, 1984, 1990, 1996, and 2002) for IMR trend in India. All the five change-points showed much higher confidence levels (CI>95%). The rate of decline in infant mortality rate was more remarkable during 1911–78. During this period, infant mortality rate dropped by 157 per 1000 live births. This pronounced phase of IMR change is majorly attributed to progress against communicable diseases such as dysentery, tetanus, polio, leprosy and absence of major famines in the post independence period [5], [38], [55]–[56]. Further, a careful observation of trends in short-term intervals indicates that there are ups and downs in the progress until 1977. Primarily due to this reason, the first critical change-point is appeared in 1978. However, more sustained critical changes in IMR that occurred in the year 1990, 1996 and 2002 were closely attributable to progress in socioeconomic conditions and steady improvement through wide-ranging mother and child health services. Commensurately, the government spending on maternal and child health programmes also increased manifold during this period [5], [38], [56]–[57].

### Population Size

India is currently the second most populous and not too far to overtake China soon to become the most populous country in the world. Of the four principal components that this study addressed in assessing the long-term trends, the eventual but a critical component of demographic transition following mortality change is the natural increase in population size. Persistent decline in infant mortality rate in the early stages of high fertility accelerates the natural increase in population size. To find critical change-points in the trends of population size of India, this study performed change-point analyses of historical trends in population size. The population size estimates of India mainly come from decadal census counts of the population. The history of the Census began in 1800 when England had begun its Census, but the population of dependencies was not known at that time. In its continuation, based on this methodology, a census was conducted in some towns and provinces of British India. Though, the first census of India began in 1871 but, the modern census started in 1881; since then the Census of India has provided uninterrupted counts of population for every ten years [5], [26], [28], [38]–[39]. In trend analysis, however, the ten year period is rather a bigger interval to assess the change-points in its entirety. It is possible that there may be changes within the ten year duration; hence, to get a fairly good picture of change-points, we have used population size estimates of five year interval based on Census population counts [43]. Similarly, Mukherjee [41] provided population size estimates for five year intervals for pre-independence India.

**Figure 3:**
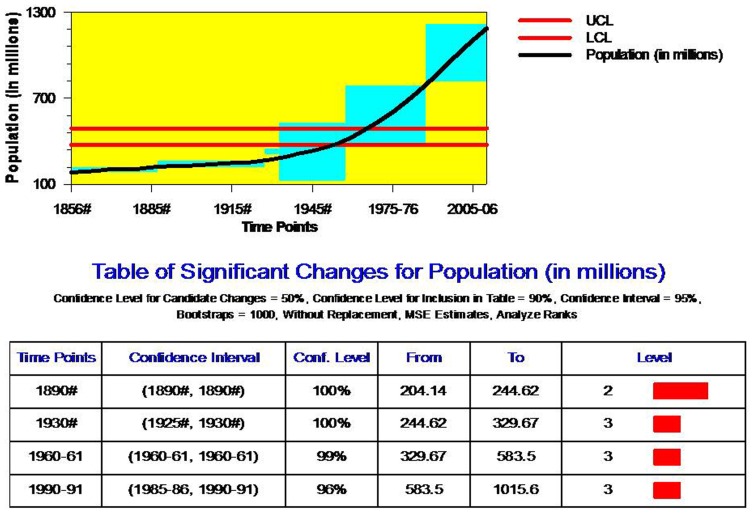
Change Point Analyses of Trend in Population Size for India, 1856–2011. Note: 1. UCL- Upper side of Confidence Level; LCL- Lower side of Confidence Level. 2. Level- Confidence Level. 3. # Estimates based on Mukherjee (1969).

The trend assessment of population size during the 1856–2011, through ‘Change-Point Analyzer’ show that, in a period of 155 years, India experienced critical changes in its population size at four time points: 1890, 1930, 1960–61, 1990–91 (figure 3). All the four change points, however, conveniently fall in census years. The change point estimates are statistically significant with above 95 per cent confidence levels. The year 1890 and 1930 shows the greater the confidence level (CI = 100%) compared to 1960–61 (CI = 99%) and 1990–61 (CI = 96%). A greater volume of change in population size (432 million) has been observed in 1990–91, followed by a change of 254 million in 1960–61. This is obvious as last two change-points were observed during India’s rapid population growth phase (1951–1991) whereas 1890 was represented the stagnant phase of population growth (prior to 1921) and 1930 was the phase of steady increase in population growth during 1921–1951 [5], [22], [25].

Available literature [3], [22], [41]–[42] advances a variety of plausible explanations that may be attributable to these four time points as a critical change point in population growth trends. The year, 1890 as a critical change-point in population size is likely due to changes in the procedures of estimation of population of India. India underwent first modern Census in 1881 for counting population size. The improved counting of the population in 1881 provided a fair chance of having a critical change in population size of India compared to its earlier estimates.

Secondly, it is again obvious to notice that 1930 as a critical change point in the trends of population size in India; as there was an important event which set as a crossroads for the preceding period of 1921. The year 1921 was called the year of the ‘Great Divide’ because it distinguished the earlier period of chequered population growth from the period of moderately increasing growth. Thirty years prior to 1921, the varying fortunes of mortality levels were responsible for variation in the growth rates of the population. The decade 1901–11 witnessed several local famines and Plague, Influenza and Malaria epidemic which caused the death of an estimated 7 percent of the total population of India [25], [39], [41]. Consequently, the growth of the Indian population was negative during the period of 1901–1911. However, in the succeeding decade (1921–31), following recovery from famines and epidemics, India experienced 28 million or more than one and a half times increase in population. The 1921 sowed the seeds of future phenomenal and accelerating growth that was witnessed during 1921–31. An accumulative growth in India’s population size was resulted in a critical change-point in 1930. The rapid growth of population in the post-1950 following a steady mortality decline is responsible for a critical-change point in population size during 1960–61. Several researchers affirm this growth was majorly driven by fall of death rate [25], [27]. The estimates of the average birth and death rates in India show that while death rates have sharply declined in each successive decade up to 1951, birth rates have virtually remained unchanged up to 1961 [26], [39], [42]. This period represented the inevitably long demographic lag period between the onset of mortality decline in 1920 and the onset of fertility decline in the 1960s. However, during the decade of 1981–91, the population of India increased by 24 percent and the average annual exponential growth rate peaked 2.14. This led to a phenomenal change in population size during 1990–91 rendering this as one of the critical change-point in the history of India’s population growth. Though, there is a considerable decline in mortality is contributed to increase in the population size.

### TFR

While the onset of infant mortality was started in post-1921 but the onset of fertility decline was in 1965. Since then fertility in India has declined and the pace of decline has accelerated from 1980s and consequently the country is currently passing through the third stage of fertility transition. Given the scale and diversity of India’s population and the provision of voluntary choice in family size norm, a decline from around six births per women in 1970 to less than half that level within a span of 30–40 years is a significant achievement [24], [26], [30]–[31], [33], [59]. However, changes in fertility across the time scale are not uniform; the decline in fertility has been highly sensitive to mortality scenarios, population-health policies and programme shifts [24], [31], [33], [59]. In general, the previous literature indicates that the year 1965–70 is determined as the period of onset of fertility decline in India; however, none of the above studies have tried to determine multiple critical changes that marked the long-term fertility trends of India based on more sophisticated tools of trend assessment. In this paper, we have not only assessed critical change-points in long-term fertility trends but also presented plausible reasons for such changes during that particular time point.

**Figure 4:**
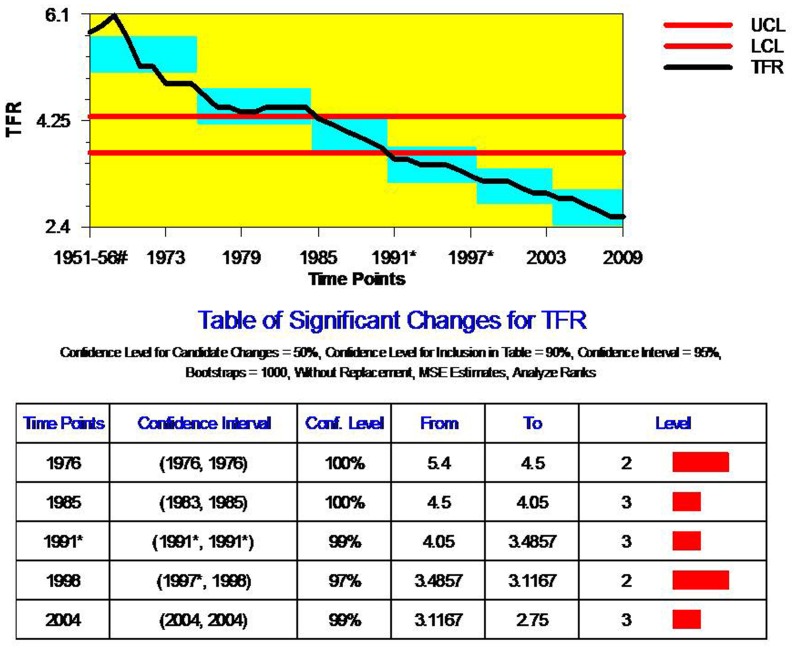
Change Point Analyses of Trend in Total Fertility Rate for India, 1951–2009. Note: 1. UCL- Upper side of Confidence Level; LCL- Lower side of Confidence Level. 2. Level- Confidence Level. 3. * Excludes Jammu & Kashmir due to non-receipt of returns. Source: 1. ^#^Rele, 1987. 2. Office of Registrar General of India, 1971–2005.

Figure 4 presents ‘Change-point analysis’ trend line plot and estimates of critical change-points for fertility rates during 1951–2009. The assessment of the trend line plot and estimate of change-points indicates that India experienced five critical change-points (1976, 1985, 1991, 1998 and 2008) during the past half a century of fertility trend. The first critical change in Indian fertility trend was observed in 1976; during this year, fertility declined by almost 20 percent from a TFR of 5.4 to 4.5 per women with a confidence level of 100 percent. The year 1976 clearly marked a turning point in India’s fertility trends because fertility remained more or less stable (or even to have increased) during the initial period until 1961–66; the estimate for the period, 1966–71 marked the true beginning of fertility decline. The onset of India’s fertility decline in 1961–66, also appears to coincide with a major change in the Indian family planning programme, from a clinic-based approach to the extension approach in the late 1963. Additionally, the establishment of a full-fledged department of Family Planning in the Ministry of Health and Family Planning in 1966 was deemed to be an important step to pursue predetermined goals of fertility decline. The establishment of this department of family planning was accompanied by a substantial increase in total expenditure for India’s family planning programme. The objective was to make family planning programme services widely and easily accessible and over time a variety of incentives were introduced and offered to acceptors of family planning programme as part of the strategy to voluntarily promote family planning and fertility control. Consequently, fertility decline accelerated, with an estimated TFR of 5.78 in 1966–71, 5.37 in 1971–76, and 4.65 in 1976–81; a decline of about one child per woman during the decade of 1971–81, or, to refer to the midpoints of this period, approximately 1975–06, the year 1976 represents the first critical change-point of fertility trend in India.

The most notable change-point in fertility trends in terms of greater volume of decline in TFR were observed in 1991. At this point of time, India experienced a decline of TFR from 4.05 to 3.48, which was the highest volume of TFR decline among all the observed change-points. The greatest decline of TFR in 1991 is closely associated with the shift in family planning and target oriented family welfare goals during the late 1970s and 1980s. However, the volume of TFR decline in other two observed change-points (1998 and 2004) was also substantial. Change-point estimates in figure 3 showed greater statistical confidence levels for all the five change-points. However, the decline in TFR during the post-1990s and an observed critical change point in 1998 or because of a change in socioeconomic status in the post-economic reform period. The recent change-point, in 2004 can be stated as the result of sizeable changes in fertility rates of traditionally high fertility states such as the Empowered Action Group (EAG) states on account of more focused and intense fertility reduction policy drive in these states and widespread diffusion of small family norms and contraception use among uneducated women. Studies have demonstrated that since the 1990s and through the period of 2000s India’s fertility decline has been driven by major fertility decline among the illiterate and poor women through widespread use of female sterilization [60]. Second, complementary since the year, 2001, the government of India has made much bigger budget allocations to improve population and health indicator of EAG states, which majorly contributed to the decline in fertility rates in these states. Overall, the pro-women and child health programmes and the population stabilization policy drive have helped in accelerating the pace of fertility decline and heading to convergence in national fertility levels [31], [33], [59].

### LEB

LEB is the most widely used aggregate mortality measures in public health research [58]. In the Indian context, many recent studies on mortality in general and LEB in particular focused on the recent trends (post-1970s) are typically based on sample registration system data. Declining infant mortality rate has been considered as a direct consequence on the improvement in overall survival times of a given population [28], [58]. However, all such studies used simple trend line plots and charts as tools for analyses. With the exception of studies that dealt with analysis of mortality patterns and their determinants, no recent study examined the long-term historical trends in the LEB. Regardless of the discrepancies in annual rates of the LEB, however, in the long-run on an average LEB level in India has more than doubled since pre-Independence days [24], [28], [26], [39]. In search of historical trends in LEB, we reconstructed the historical change points in LEB and quantified the magnitude of change-points based on robust methodological tools.

**Figure 5:**
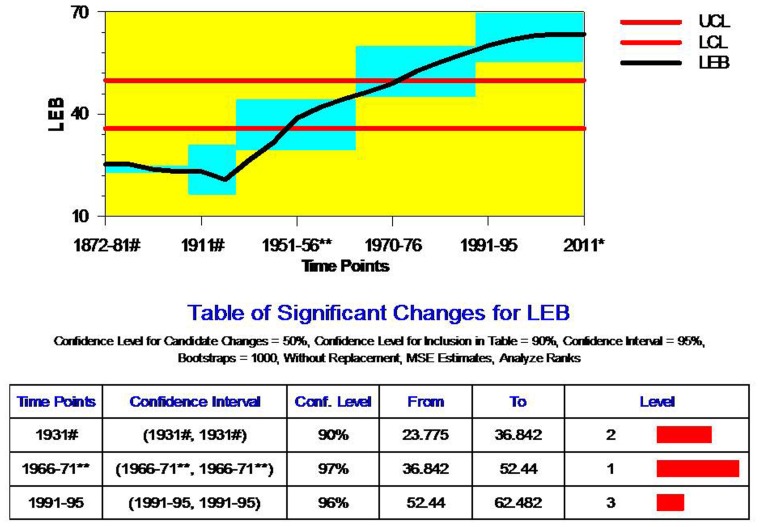
Change Point Analyses of Trend in Life Expectancy at Birth for India, 1872–2011. Note: 1. UCL- Upper side of Confidence Level; LCL- Lower side of Confidence Level. 2. Level- Confidence Level. Source: 1. ^#^ Mitra, 2005 documented LEB estimates based on Registrar General of India and Census during 1872–1941. The estimates presented in the table are male female averages. 2. **Rele, 1987. 3. Office of Registrar General of India, 1970–2006. 4. *UNDP, 2011.

Figure 5 shows change-point analyses for LEB trends during 1871–2011. The results unravel three critical change-points (1931, 1966–71, 1991–95) for LEB over the period of 140 years. All the three change-points show high confidence levels (CI>90%). However, the long-term trend in India revealed a greater change in LEB during 1966–71 with greater confidence level (CI>97%). The patterns of critical change-point in LEB are seen also closely tied to epidemics and famines, medical and mortality scenarios and major changes in socioeconomic conditions in India. The critical change in LEB in 1931 was mainly due to the spectacular progress in food supplies, health care provisions to control epidemics and communicable diseases after dreadful famines during 1901–11; thereafter it was followed by a recovery period. The graphical presentation of trend line in figure 4 also shows that, the rate of decline in LEB in the post-1931 period slowed down after 1950 but regained progress ever since 1960. The progress following slowdown is consistent with the swift reduction in infant mortality rate and further reductions in adult mortality rates in the post independence period. The accelerated increase in LEB in the post-1960 marked a critical change-pint in 1966–71. This is the period where India experienced sustained decline in death rates that lead to pronounced increases in the LEB. However, the major changes in LEB in 1991–95 are attributed to fall in IMR, improvement in socioeconomic and health status which further lengthened the life span in India. The death rate in India declined significantly from 27 per thousand in 1941–50 to 11 per 1000 population in 1986–91. During this period, India improved in terms of maternal and child health which is also greatly contributed to increase in the LEB.

### Conclusion

Although, demographic transition is quite a familiar idea during the past half century period, it was regarded more an abstraction than a description of the true trajectory of population and health indicators; because demographic statistics hardly go back to the beginning of the cycle and there is no certainty that when the end of it will be reached in developing countries like India. According to the current demographic scenario, it is hard to determine the end point of demographic transition in India. However, over the past half century, the progress in fertility and mortality decline in India is remarkable [24], [26], [34], [39], [61]. Several past studies, which examined the process of demographic transition in India, recognized very few major shifts while concluding that overall demographic trends in India are transitioning from third to the fourth stage of demographic transition [5], [27], [29], [30]–[36]. In view of considerable analytical limitations of such previous studies, in this study we have advanced the ‘change-point analyses’ as a new methodological tool for assessment of progress and changes in population and health indicators for the larger goal of understanding the true trajectories of the population and health transition.

The findings of this study foster that, the trajectory of long-term demographic trends in India, in four key demographic indicators, resulted in several major shifts. ‘Change-point analyses’ for IMR, Population size, TFR and LEB reveal multiple critical changes over a long-run period of demographic history in India. The shifts in IMR and TFR trends are closely associated with strategic shifts in the approach of the family welfare programme in India. However, the critical change-points observed in pre-independence period for Population size and LEB are majorly attributed to fluctuations in these indicators emerged out of famines, several communicable diseases and epidemics. A sustainable and the continued demographic transition was initiated during the mid - 1960s. Therefore, the causal linkages among four major components of demographic transition are interpreted only for the trends observed in post-1965.

In the Indian context, this study fosters that transition in fertility is initially followed by infant mortality. As evident from our trend line graphs, the infant mortality steeply declined ever since 1921 but fertility transition was initiated in post-1965. The first critical change-point in fertility emerged two years before the infant mortality is basically attributed to 1) Though, the volume of infant mortality decline during a larger interval (1921–1977) was high but ups and downs in the progress in short-intervals, delayed the emergence of first critical change-point in infant mortality until 1978. 2) On the other hand, targeted and forced family planning programme during this period facilitated advantage for a sustained decline in TFR. However, in the later periods, the other critical change-points in infant mortality appeared earlier than critical change-points in fertility. Again, this is due to a sustained decline in infant mortality in post-1978 and change in approach of family planning programme in a later phase of 1975. Overall, the improvement in socioeconomic conditions, family planning and maternal and child health services are the driving force of the decline in child mortality and fertility after mid-1970s. Trends in population size experienced most critical change-point in 1990–91. It took hundreds of years to reach first 500 million populations, however, the second 500 million populations were added to the total population size (1186 million) in just 40 years (from 1971 to 2011). A critical change in LEB during 1991–95 is clearly attributable to decline in mortality rates during 1970–1990 with improved health care provisions and socioeconomic conditions.

Overall, this study advanced a number of critical insights on demographic transitional change-points connecting it to contextual policy shifts in Indian context. The evolutionary construction and interpretation of long-run demographic trends and the vital demographic change-points in this study have certainly enhanced our understanding of trends of population and health indicators vis-a-vis population and health policy shifts. Critical change-points in population and health indicators in India are associated with the evolution of structural changes in population and health policy framework. The results have not only helped in reassessing the historical past and the current demographic transition trajectory but also advanced a new statistical tool for assessing the demographic trends that are necessary for robust monitoring of the progress in population and health trends. There are no specific limitations with change-point analyzer tool. However, it will provide the best results with data of a long period of time. Nevertheless, this study has a limitation in terms of data used: 1) in India, we do not have a long term data from a single source of official statistics; therefore, we are compelled to use multiple data sources for this study. 2) The demographic estimates before the 1950s were mostly based on indirect estimates.

## Supporting Information

Appendix S1 **CUSUM Charts of Long-Term Trends of Selected Population and Health Indicators of India, 1872–2011.**
(TIF)

## References

[1] Notestien FW (1945) Population: the long view. In SCHULTZ ed. Food for the world. Chicago: University of Chicago Press.

[2] Sinding SW (2001) Foreword. In Bulatao RA, Caterline JB, eds. Global Fertility Transition, Population and Development Review, A supplement to Vol. 27: 9–10.

[3] Davis K (1951) The population of India and Pakistan. Princeton: Princeton University Press.

[4] Davis K (1963) The theory of Change and Response in Modern Demographic History, Population Index. 29: 345–366. 12335951

[5] Dyson T (2004) India’s population - the future. In Dyson T, Robert C, Visaria L, eds. 21st Century India: Population, Environment and Human Development. Oxford: Oxford University Press.

[6] Dyson T (2010) Population and development: the demographic transition, Zed Book publication, London and New York.

[7] Casterline JB (2011) Review of Tim Dyson’s Population and Development: The Demographic Transition. Population and Development Review 37(2): 395–397. 10.1111/j.1728-4457.2011.00377.x 21280364

[8] Van De Walle E, Knodel J (1980) Europe’s fertility transition: new evidences and lessons for today’s developing world. Population Bulletin 34(6): 3–44. 12309786

[9] Bloom DE, Williamson JG (1998) Demographic transition and economic miracles in emerging Asia. World Bank Economic Review 12: 419–55.

[10] Wilson C, Airey P (1999) How can a homeostatic perspective enhance demographic transition theory. Population Studies 53(2): 117–128.

[11] Casterline JB (2001) The pace of fertility transition: national patterns in the second half of the twentieth century. Population and Development Review 27: 17–52.

[12] Wilson C (2001) On the scale of global demographic convergence 1950–2000. Population Development Review 27: 155–71. 18589488 10.1111/j.1728-4457.2001.00155.x

[13] Wilson C (2011) Understanding global demographic convergence since 1950. Population Development Review 37(2): 375–88. 10.1111/j.1728-4457.2001.00155.x 18589488

[14] Bongaarts J (2003) Completing the Fertility Transition in the Developing World: The Role of Educational Differences and Fertility Preferences. Working Paper, Policy Research Division, New York: Population Council. 10.1080/0032472032000137835 14602532

[15] Weeks JR, Getis A, Hill AG, Gadalla S, Rahed T (2004) The fertility transition in Egypt: intra-urban pattern in Cairo. Annals of the Association of American Geographers 94(1): 74–93.

[16] Caldwell JC (2004) Demographic theory: A long view. Population and Development Review 30(2): 297–316.

[17] Moser K, Shkolnikov V, Leon D (2005) World mortality 1950–2000: divergence replaces convergence from the late 1980s. Bulletin of the World Health Organization 83(3): 202–209. 15798844 PMC2624202

[18] Doepke M (2005) Child mortality and fertility decline: Does the barro-becker model fit the facts? Journal of Population Economics 18: 337–366.

[19] Dorius SF (2008) Global demographic convergence? a reconsideration of changing inter-country inequality in fertility. Population and Development Review 34: 519–537.

[20] Dorius SF, Firebaugh G (2010) Trends in global gender inequality. Social Forces 88(5): 1941–1968. 10.1353/sof.2010.0040 PMC3107548 21643494

[21] Angeles L (2010) Demographic transitions: analyzing the effects of mortality on fertility. Journal of Population Economics 23(1): 99–120.

[22] Ghosh A (1956) Demographic trends in India during 1901–50. Population Studies 9(3): 217–36.

[23] Rele JR (1982) Trends and differentials in fertility in population of India. Country Monograph Series No.10, ST/ESCAP/220. New York: ESCAP, U.N.: 91–108.

[24] Rele JR (1987) Fertility levels and trends in India, 1951–81. Population and Development Review 13(3): 513–530.

[25] Dyson T (1989) The population history of Berar since 1881 and its potential wider significance. Indian Economic and Social History Review 26(2): 167–201.

[26] Bhat PNM (1989) Mortality and fertility in India, 1881–1961: a reassessment. In Dyson T, ed. India’s Historical Demography: Studies in Famine, Disease and Society. London: Curzon Press.

[27] Bhat PNM, Rajan SI (1997) Demographic transition since independence. In Zachariah KC, Rajan SI, eds. Kerala’s Demographic Transition: Determinants and Consequences, India. New Delhi: Sage Publications, 33–78.

[28] Bhat PNM, Preston SH, Dyson T (1984) Vital rates in India, 1961–1981, Committee On Population And Demography, Report no.24. National Academy Press, Washington D.C.

[29] James KS (1995) Demographic transition and education in Kerala. Economic and Political Weekly 30(51): 3274–76.

[30] Guilmoto CZ, Rajan SI (2001) Spatial pattern of fertility transition in Indian districts. Population and Development Review 24(4): 713–738.

[31] Visaria P, Visaria L (1994) Demographic transition: accelerating fertility decline in 1980s. Economic and Political Weekly 29(51/52): 3281–3292.

[32] Visaria P, Visaria L (2003) Long-Term Population Projections for Major States, 1991–2001. Economic and Political Weekly 38(45): 4763–4775.

[33] Kulkarni PM, Alagarajan M (2005) Population growth, fertility and religion in India. Economic & Political Weekly 45(5): 403–11.

[34] Visaria L (2004b) The continuing fertility transition. In Dyson T, Cassen R, Visaria L, eds. Twenty-First Century India–Population, Economy, Human Development, and the Environment (pp 57–73). New Delhi: Oxford University Press.

[35] Kulkarni PM (2011) Towards an explanation of India’s fertility transition. Paper presented at the George Simmons Memorial Lecture, 33rd Annual Conference of the IASP, Lucknow, November 11–13, 2011.

[36] Visaria L (2011) Demographic transition in South India, Special Series Paper, Population Foundation of India, New Delhi.

[37] Taylor W (2011) Change-point analysis: a powerful new tool for detecting changes. Taylor Enterprises, Libertyville, Illinois. Available online at: http://www.variation.com/cpa.

[38] Chandrasekhar S (1972) Infant mortality, population growth and family planning in India, Routledge, London.

[39] Office of Registrar General of India (2009) Compendium of Sample Registration System. Registrar General of India, Ministry of Home Affairs, Government of India.

[40] United Nations Development Programmeme (2011) Human Development Index Report-2011. India, New York, USA.

[41] Mukherjee M (1969) National income of India: trends and structure. Statistical Publishing Society, Calcutta.

[42] Mahalanobis PC, Bhattacharya D (1976) Growth of population in India and Pakistan-1801–1961. Artha Vijnana 18: 1–10.

[43] RBI (1955–2011) Handbook of statistics. New Delhi: Reverse Bank of India, Government of India.

[44] Census of India (1871–2001) Final population totals. New Delhi: Office of the Registrar General of India.

[45] Census of India (2011) Provisional population totals. New Delhi: Office of the Registrar General of India.

[46] Page ES (1955) A test for a change in a parameter occurring at an unknown point. Biometrika 42: 523–527.

[47] Page ES (1957) On problems in which a change in parameter occurs at an unknown point. Biometrika 44: 248–252.

[48] Hinkley DV (1971) Inference about the Change-Point from Cumulative Sum Tests. Biometrika 58(3): 509–523.

[49] Hinkley D, Schechtman E (1987) Conditional bootstrap methods in the mean-shift model. Biometrika 74(1): 85–93.

[50] Pettitt AN (1980) A simple cumulative sum type statistic for the change-point problem with zero-one observations. Biometrika 671: 79–84.

[51] Box G, Luceño A. (1997) Statistical control by monitoring and feedback adjustment, Wiley, New York.

[52] Sibanda T, Sibanda N (2007) The CUSUM chart method as a tool for continuous monitoring of clinical outcomes using routinely collected data. BMC Medical Research Methodology 7(46): 1–7. 17980042 10.1186/1471-2288-7-46 PMC2204022

[53] Fujisaki I, Pearlstine VE, Miller M (2008) Detecting population decline of birds using long-term monitoring data. Population Ecology 50: 275–284.

[54] Taylor W (2000a) Change-Point Analyzer 2.0 Shareware Programme, Taylor Enterprises, Libertyville, Illinois. Available online at: http://www.variation.com/cpa.

[55] Karkal M (1985) Maternal and infant mortality: reviewed Work(s). Economic and Political Weekly 20(43): 1835–1837.

[56] Efron B, Tibshirani R (1993) An introduction to the bootstrap, Chapman & Hall, New York.

[57] Dasgupta M (2005) Public health in India: Dangerous neglect, reviewed work(s). Economic and Political Weekly 40(49): 5159–5165.

[58] Srinivasan K. Chander Shekhar, Arokiasamy P (2007) Reviewing reproductive and child health programmemes in India. Economic & Political Weekly 42(27): 2931–39.

[59] Preston SH (1980) Causes and consequences of mortality declines in less developed countries during the twentieth century. In Easterlin RA, ed. Population and Economic Change in Developing Countries (pp. 289–360). Chicago and London: The University of Chicago Press.

[60] James KS, Nair SB (2005) Accelerated decline in fertility in India since the 1980s: trends among Hindus and Muslims. Economic & Political Weekly 45(5): 375–84.

[61] McNay K, Arokiasamy P, Cassen RH (2003) Why are uneducated women in India using contraception? a multilevel analysis. Population Studies 57(1): 21–40. 12745807 10.1080/0032472032000061703

