# Supplementary figures and images for: Demographic Transition in India: An Evolutionary Interpretation of Population and Health Trends Using ‘Change-Point Analysis’
Source: PLoS One. 2013 Oct 18;8(10):e76404. doi: 10.1371/journal.pone.0076404 (PMC3799745; doi:10.1371/journal.pone.0076404)

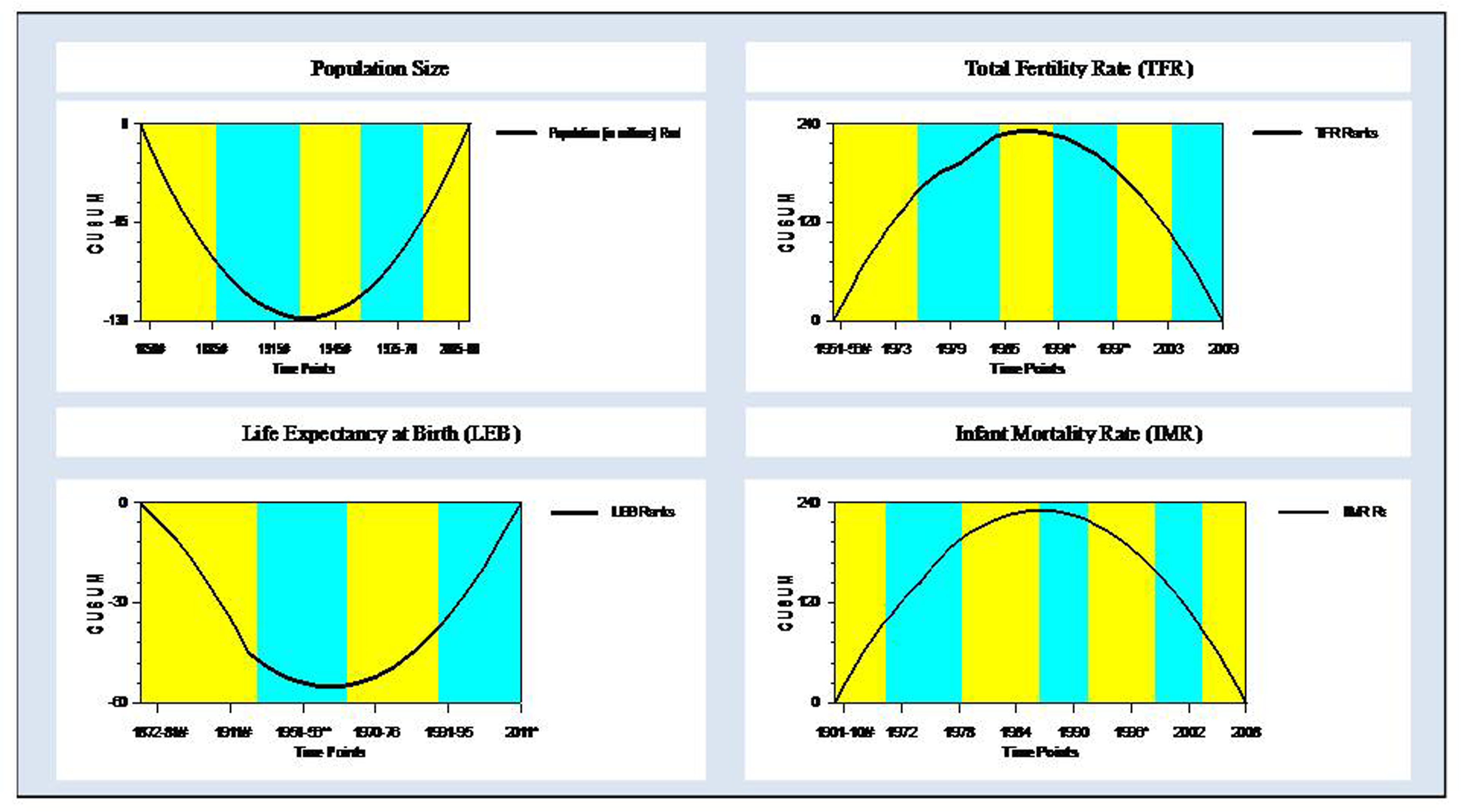

Supplement: Appendix S1 — CUSUM Charts of Long-Term Trends of Selected Population and Health Indicators of India, 1872–2011. (TIF) [file pone.0076404.s001.tif]
